# Usefulness of a Mobile Application (Mentali) for Anxiety and Depression Screening in Medical Students and Description of the Associated Triggering Factors

**DOI:** 10.3390/brainsci12091223

**Published:** 2022-09-10

**Authors:** Margarita L. Martinez-Fierro, Anayantzin E. Ayala-Haro, Martha E. Pinedo-Hurtado, Jorge A. Solis-Galvan, Idalia Garza-Veloz, Zihomara Y. Velazquez-Lopez, Antonio G. Camacho-Martinez, Lorena Avila-Carrasco, Sodel Vazquez-Reyes, Perla Velasco-Elizondo, Alejandro Mauricio-Gonzalez, Yolanda Ortiz-Castro

**Affiliations:** 1 Molecular Medicine Laboratory, Academic Unit of Human Medicine and Health Sciences, Universidad Autónoma de Zacatecas, Carretera Zacatecas-Guadalajara Km.6. Ejido la Escondida, Zacatecas 98160, Mexico; 2 Academic Unit of Electrical Engineering, Universidad Autónoma de Zacatecas, Carretera Zacatecas-Guadalajara Km.6. Ejido la Escondida, Zacatecas 98160, Mexico

**Keywords:** affective disorders, anxiety, depression, Beck inventories, psychology, psychiatry

## Abstract

The impact of the COVID-19 health crisis on the mental health of the population requires the implementation of new primary screening strategies of mental health disorders to intervene in a timelier manner, and technology may provide solutions. We aimed to evaluate the usefulness of the mobile app Mentali (version 1.1.2; creators: Jorge Alfonso Solís Galván Sodel Vázquez Reyes, Margarita de la Luz Martínez Fierro, Perla Velasco Elizondo, Idalia Garza Veloz, Alejandro Mauricio González and Claudia Caldera Villalobos, Zacatecas, México) as a primary screening tool for anxiety and depression disorders in medical students and to assess the triggering risk factors. This was a descriptive and longitudinal study and included 155 Mexican medical students. Participants interacted with Mentali for 6 months. The mobile app integrated the Beck anxiety and depression inventories together with a mood module. At the end of the interaction, the students received psychological and psychiatric interventions to confirm their primary diagnoses. Symptoms of moderate/severe anxiety and depression were present in 62.6% and 54.6% of the studied population. When corroborating the diagnoses, Mentali obtained a sensitivity of 100%, 95%, and 43% to classify a mental health disorder, anxiety, and depression, respectively. The most important triggers found were as follows: belonging to a dysfunctional family, being introverted, and having suffered from bullying. The proportion of users with excellent/good mood decreased from 78.7% to 34.4% at the end of the semester, and the proportion of users who claimed to have bad/very bad mood increased from 7.4% to 34.4% at the end of the semester (*p* < 0.05). Mentali was useful for identifying users with anxiety and/or depression, and as an auxiliary tool to coordinate the provision of specialized interventions, allowing us to increase the proportion of patients who needed psychological care and received it by 30%. The efficacy of Mentali in identifying activities through time with an impact on the mood and mental health of the users was confirmed. Our results support the use of Mentali for the primary screening of mental health disorders in young adults, including medical students.

## 1. Introduction

Anxiety and depression are problems with a great impact on public health [1]. The importance of these problems has increased in recent years due to the COVID-19 pandemic, which began with an outbreak reported in Wuhan in China on 31 December 2019 [2]. Worldwide, according to the World Health Organization (WHO) data, during the first year of the COVID-19 pandemic, the prevalence of anxiety and depression experienced an increase of 25% [3], and depression was considered a leading cause of disability [2]. In Mexico, the National Survey of Self-Reported Well-Being (ENBIARE) in 2021 reported that the proportion of the population with symptoms of depression and anxiety amounted to 15.4% and 31.3% of the adult population, respectively [4].

The Diagnostic and Statistical Manual of Mental Disorders, Fifth Edition (DSM-5) considers depressive disorders as a disruptive deregulation of mood, with common features of its subdivisions including the presence of sadness, irritability, or feelings of emptiness [5]. Depressive disorders may be associated with disabling somatic and cognitive changes, differing in presentation, duration, and possible etiology, where major depressive disorder is the classic presentation, characterized by episodes with a duration of at least 2 weeks [5]. Regarding anxiety disorders, they share the characteristics of excessive anxiety and fear, understanding fear as an emotional response to an imminent threat, real or imaginary, and anxiety as an anticipatory response to a future threat [5,6]. These conditions differ from each other by the situations or objects that induce them, the type of associated cognition, and the duration. Anxiety and/or depression may begin in childhood and persist if they are not treated, or they may also be established in adulthood [5]. Mental health disorders represent an enormous cost to society [7], and, in individuals with these conditions and without treatment, they have been associated with other pathologies, such as fibromyalgia, thyroid disorders, and arterial hypertension [8], as well as suicide, which has a strong link [9].

In Mexico, the recent COVID-19 Health Care Workers Study (HEROES), promoted by the Pan-American Health Organization (PAHO) [10], reported that 59.6% of Mexican health personnel had symptoms of mild depression, 24% had moderate symptoms, 10.6% were diagnosed with moderate to severe depression, and 5.7% showed severe symptoms of depression [11]. Similarly, among young adults who are pursuing university studies, medical students experience higher levels of anxiety due to the high academic demands [12]. Pre-pandemic studies have shown that 1 in 3 medical students suffer from anxiety, with an overall prevalence of 33.8%, and moderate to severe depression in 14%. Despite extensive studies, there are no clear common risk factors associated with this mental condition and therefore more studies are needed to identify them [13,14,15].

Recently, the potential of mental health apps to improve the monitoring and management of mental health disorders [16] has emerged, but the development of a specific, brief screening test that facilitates the identification of at-risk individuals is necessary [17]. Recent studies have made it clear that the status of mental health in the population can be evidenced by internet searches [18]; however, there is currently insufficient evidence to support the effectiveness of apps for mental health screening and/or classification, and therefore methodologically sound research studies evaluating their effectiveness are needed [19,20,21]. The usefulness or diagnostic capacity of a test is conferred by the presence/absence of the disease in the individuals to whom it is applied and actually have or do not have the disease, where the sensitivity and specificity are the parameters that define its validity, and these are considered intrinsic characteristics of the test [22,23]. Currently, there is a lack of technological tools used for the screening of mental health disorders with a follow-up by professionals [24], which may support and validate their diagnostic capacity [25]. The use of mobile applications is a promising area, since it allows optimizing the time of attention, the immediacy of data processing and resolution, a friendly design, and confidentiality and cost-effectiveness [26,27,28].

### Objective

The aim of this study was to evaluate the usefulness of a mobile app (Mentali) as a primary screening tool for anxiety and depression disorders in Mexican medical students. In addition, we also evaluated the main factors associated with the development of mental health problems in the studied population.

## 2. Materials and Methods

### 2.1. Study Design and Participants

This was a descriptive and longitudinal study carried out in Zacatecas, Mexico. First-semester medical students from the Academic Unit of Human Medicine and Health Sciences of the Autonomous University of Zacatecas were invited to participate voluntarily. After providing informed consent, all the participants were invited to download and interact with the Mentali app. There were no exclusion criteria for the study. Users who did not interact with Mentali or who decided to abandon the project were eliminated from the study. The recruitment was conducted from August to December 2021.

### 2.2. Instruments and Scales

#### 2.2.1. Mentali App

Mentali is a mobile application developed by the team of the Molecular Medicine Laboratory from the Universidad Autónoma de Zacatecas, in Mexico (creators: Jorge Alfonso Solís Galván, Sodel Vázquez Reyes, Margarita de la Luz Martínez Fierro, Perla Velasco Elizondo, Idalia Garza Veloz, Alejandro Mauricio González, and Claudia Caldera Villalobos. Registration number: 03-2021-121414575000-01). It was designed for the primary screening of anxiety and depression. Mentali is available in the App Store (https://n9.cl/mentaliapp (accessed on 1 July 2021)), Play Store (https://n9.cl/mentali_playstore (accessed on 1 July 2021)), and App Gallery (https://n9.cl/mentali_appgallery (accessed on 1 July 2021)). The app consists of 3 modules: 2 concerning the Beck inventories for anxiety and depression, respectively, which allow responses in two-week intervals, and another module consisting of a traffic light mood graph, which can be answered in an unlimited manner with the answers “Excellent”, “Good”, “Regular”, “Bad”, and “Very bad”. The mood chart provides the option to freely write about the activities performed when answering. The recording of the words generates a semantic field of frequent words that serves to identify the triggering factors, where they correlate with another group of words associated with a degree of severity previously established within mild, moderate, and severe categories, as shown in Supplementary Materials Table S1.

#### 2.2.2. Beck Inventories

The Beck inventories for anxiety and depression are composed of 21 items each, which allow the evaluation of the corresponding symptoms presented by the participant during the last 2 weeks [29]. These inventories have already been validated in the Spanish language [30]. The weighting of the items ranges from 0 to 3; 0 is absolute, 1 slight, 2 moderate, and 3 severe, which, when added, can yield a total score between 0 and 63 [31], thus establishing the following cut-off points:

- Anxiety: 0–7 points, minimal anxiety; 8–15, mild anxiety; 16–25, moderate anxiety; 26–63, severe anxiety.
- Depression: 0–9 points, minimal depression; 10–16, mild depression; 17–29, moderate depression; 30–63, severe depression.

### 2.3. Psychological Intervention and Clinical History

The continuous interaction of the students with the app allowed us to automatically determine the “alerts”, which were equivalent to a moderate–severe score on 2 consecutive occasions. This measure was applicable to either inventory. Alerts within the mood module were determined manually, according to alarm words categorized as “very high” and “high” consecutively (see Supplementary Materials Table S1). Participants who generated the alerts were contacted to offer them psychological care and they were free to decide whether they were interested in receiving it. Psychological diagnosis was used as a gold standard to compare the results of the primary screening obtained using Mentali.

During the stage of intervention, both clinical and psychological history were included to produce a comprehensive diagnostic impression. At this time, the Hamilton inventories for both anxiety and depression were considered (see below for the description of these inventories). The clinical history consisted of the following sections: personal information, habitus exterior, DSM-5 criteria for major depressive disorder, current medical status, psychodrama, family history, triggers, personal assessment, pathological and non-pathological personal history, conclusions, and diagnostic impression.

In addition, as part of the psychological evaluation, the family APGAR was also included, which evidenced the way in which a person perceives the functioning of his or her family at a given moment [32]. This evaluation has shown a strong association with mental disorders in medical students [33]. Each of the established options had a score ranging from 0 to 4, where 0 = Never, 1 = Almost Never, 2 = Sometimes, 3 = Almost Always, 4 = Always. According to the above, the following cut-off points were established: 17–20, 16–13, 12–10, and <9, for normal, mild dysfunction, moderate dysfunction, and severe dysfunction, respectively.

#### 2.3.1. Hamilton Anxiety Rating Scale (HARS)

The HARS is a psychological questionnaire with a Likert-type scale that is administered by a clinician after an interview. Each item is scored from 0 to 4 points, assessing both its intensity and frequency, and 2 scores can be obtained that correspond to psychic anxiety (items 1–6 and 14) and somatic anxiety (items 7–12 and 13). A higher score indicates a higher intensity of anxiety, with the maximum score being 56. The HARS is sensitive to variations over time or after treatment. The cut-off points are as follows: <17, 18–24, 25–30, and >31, for minor, moderate, severe, and very severe anxiety [34,35], respectively.

#### 2.3.2. Hamilton Depression Rating Scale (HDRS)

The HDRS is a psychological test that uses a Likert-type scale, designed to be used in patients previously diagnosed with depression, with the aim of quantitatively evaluating the severity of symptoms and evaluating the changes in the patient; its updated version was created with 17 items, which is recommended by the National Institute of Mental Health of the United States [36]. Each question has between 3 and 5 answers, with a score of 0–2 or 0–4, respectively, where the total score ranges from 0 to 52. The following cut–off points have been established: 0–7 = not depressed, 8–13 = mild depression, 14–18 = moderate depression, 19–22 = severe depression, >23 = very severe depression.

After psychological evaluation, patients who needed psychiatric care were detected to provide them with joint care with pharmacological treatment if necessary, according to the psychiatric manual guidelines and the Mexican clinical practice guidelines [37,38,39]. Once the psychiatric clinical history was obtained, the diagnostic impressions of the 3 levels of care were compared.

### 2.4. Data Analysis

The information obtained during the study was stored in a Microsoft Excel spreadsheet. The data were represented as mean ± standard deviation (SD) or percentage. Comparisons of proportions between groups were carried out using Chi-square, Fisher’s exact test, and odds ratios. To evaluate the usefulness of the Mentali app to classify anxiety and depression, the Bayes theorem [22] was used. For this, 2 × 2 tables were constructed to calculate the sensitivity, specificity, positive predictive value (PPV), and negative predictive value (NPV). Sigma Plot 12.0 software (Systat Software Inc., San Jose, CA, USA) was used to perform data analysis. *p*-values < 0.05 were considered significant. For the production of graphs, Numbers v11.1 (Apple Inc., Cupertino, CA, USA), Canva web (CANVA PTY LTD, Perth, Western Australia), and the Mentali app were used.

### 2.5. Ethical Considerations

The protocol was approved by the following Institutional Review Boards: Comité de Ética en Investigación (ID: CAICCL/REV.1/03/2021) and Comité de Investigación (ID: AL-FAMC/CI/02/2020). The participation of the students was voluntary and they were included after obtaining informed consent. During the study, personal identity data were kept confidential, in accordance with the regulations of the Mexican General Health Law, the Mexican guidelines NOM-004-SSA3-2012 of the clinical record and the ethical principles of the Helsinki Declaration [40,41].

## 3. Results

### 3.1. Description of the Characteristics of the Sample

**Figure 1:**
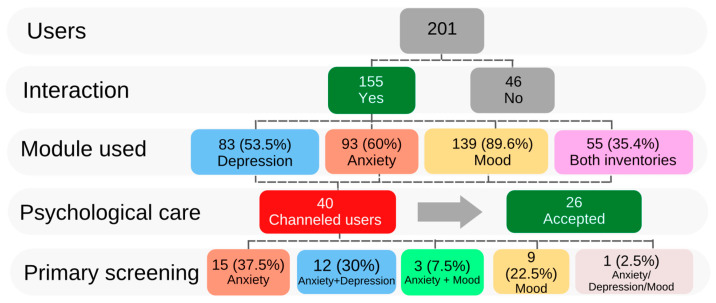
Summary of study population and results of the primary screening approach using Mentali mobile application. There were 201 participants who downloaded and installed the Mentali app; 46 of them were eliminated from the study because they did not interact with Mentali and therefore the final number of users considered for the study was 155. The diagram displays interactions of the users with each Mentali module (anxiety and depression inventories and mood), the number of users who needed psychological intervention, and the results of the primary screening.

A total of 201 first-semester medical students, who agreed to participate in the study, downloaded and installed the Mentali app on their cellphones. However, 46 students were eliminated from the study because they had no interaction with the app during the period of time evaluated. According to the above, 155 participants who interacted with Mentali were included (Figure 1). The average age was 21.3 (±1.7) years (range: 17–26 years) and 64.5% of the participants were women. A total of 121 (78%) Mentali users interacted at least once with the Beck inventories module, and 139 (89.6%) routinely interacted with the mood module. Figure 1 shows a breakdown of the first stage of the study, from the initial interaction with the Mentali app to the end of the follow-up, and summarizes the results of the primary screening.

### 3.2. Anxiety and Depression Primary Screening

**Figure 2:**
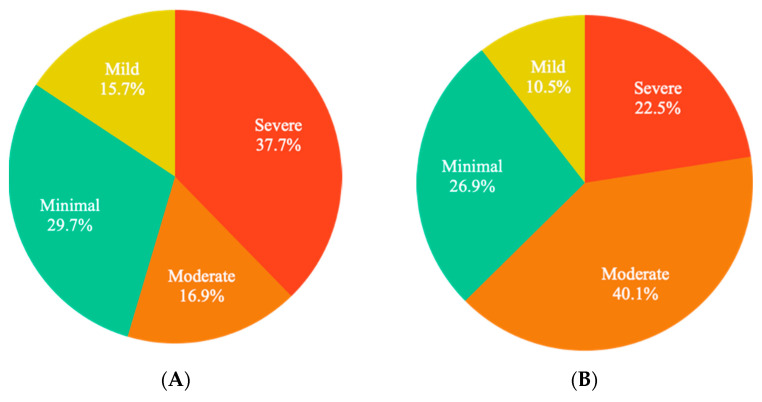
General frequency of anxiety and depression obtained during the study and its classification by severity. Figure 2 shows the results of primary screening using Mentali: (**A**) for depression (*n* = 83); (**B**) for anxiety (*n* = 93). During the period evaluated, the presence of symptoms of moderate–severe anxiety and depression were present in 62.6% and 54.6% of the studied population.

Of the 155 Mentali users included in the study, 55 (35.4%) medical students responded to both the anxiety and depression inventories. Overall, 83 (53.5%) responded to the Beck inventory of depression and 93 (60%) to the Beck anxiety inventory (Figure 1). Figure 2 shows the results of the general primary screening using Mentali, classifying patients by severity according to the Beck inventories’ criteria. During the period of time evaluated, the primary screening of moderate–severe depression was positive for 54.6% of the participants completing the inventories in the Mentali app (Figure 2A), and 62.6% had a positive test result for moderate–severe anxiety (Figure 2B).

According to the results obtained using Mentali, 40 users generated alerts and therefore were candidates for psychological intervention (Figure 1). Among these, 15 (37.5%) had a positive primary screening for anxiety, 12 (30%) for both disorders, 3 (7.5%) for anxiety plus mood, 1 (2.5%) generated an alert in all 3 modules (anxiety, depression, and mood), and 9 (22.5%) generated alerts only in relation to mood. Considering only the participants who needed psychological intervention, 26 (65%) accepted it, 12 (30%) did not respond to the invitation, and 2 (5%) participants were already receiving psychological care with an external specialist. Ten (38.4%) of the students who agreed to receive care from a specialist had obtained a positive screening for anxiety, 8 (30.7%) for both disorders, 3 (11.5%) for anxiety plus mood, 1 (3.8%) for both disorders and mood, and 4 (15.3%) had only alerts for mood.

### 3.3. Evaluation of Mood

This module was developed to identify triggering factors associated with mood and to correlate the influence of user activities on depression and/or anxiety episodes. There were 139 users interacting with the mood module (Figure 1). Among these, for 62 (40%) participants, this was the only module that they used, yielding a total of 2845 responses. Moreover, 23% of the responses for mood were “Excellent”, 35.1% “Good”, 24.1% “Fair”, 12.1% “Bad”, and 5.4% “Very bad”. When the analysis of the associated activities was performed, the words most present were “Homework”, with 385 responses, “Anatomy”, with 120, “Study”, with 250, and “Classes”, with 545. Considering this tool, nine users generated alerts and therefore psychological care was offered.

### 3.4. Psychological Care

The patients referred to psychological care (*n* = 26) comprised 18 (69.2%) women and 8 (30.7%) men. 92.3% of them were single and 84.6% were of the Catholic religion.

#### 3.4.1. Psychological Medical History

**Figure 3:**
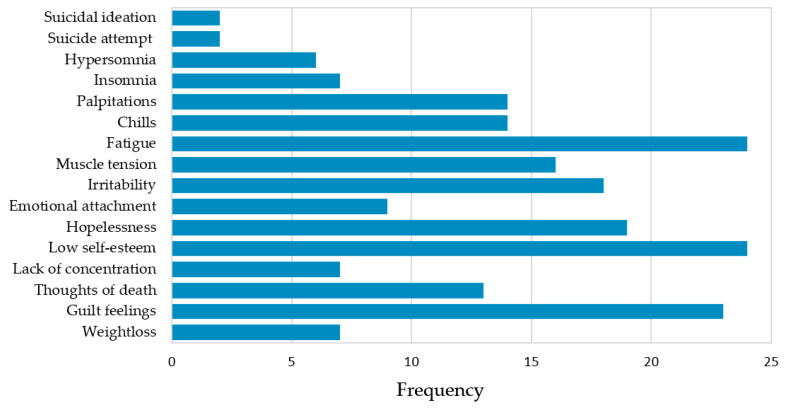
Frequency of symptoms associated with major depressive disorder and generalized anxiety disorder, according to the DSM-5. Figure 3 shows the most frequent DSM-5 criteria for major depressive disorder collected in the psychological history of the participants (n = 26). Low self-esteem, lack of concentration, and fatigue had a frequency of 92.3%.

The following results were obtained from the analysis of the external habitus of the individuals studied: 17 (65.3%) of the 26 patients had an age corresponding to their physical appearance, 9 (34.6%) had a high height, and 6 (23%) low; in relation to weight, 12 (46.1%) had a low weight and 6 (23%) were overweight. Regarding personal arrangement, 24 (92.3%) were classified as aligned. Regarding eye contact, 7 (26.9%) avoided it, and regarding behavior, 11 (42.3%) were tense and 1 (3.8%) behaved evasively; the rest of the participants were within standard ranges. When analyzing the DSM-5 criteria for major depressive disorder and generalized anxiety disorder in the study population, feelings of guilt (88%), low self-esteem (92.3%), lack of concentration (92.3%), and fatigue (92.3%) were the variables with higher frequency among the patients in psychological care (Figure 3).

In the family history section, the analysis of the family APGAR showed that 69.2% of the patients had a normal score; 3.8%, 11.5%, and 11.5% had mild, moderate, and severe dysfunction, respectively. Among the family types, 73% of the patients had a nuclear family, 15.3% had single-parent families, 7.6% reconstructed, and 3.8% of the participants did not answer. The psychological history also collected the risk factors by social area (Table 1), where the presence of conflicts in the family (46.1%) and in the relationship (15.3%) and school stress (50%) predominated; likewise, the triggers by life stages were evaluated (Supplementary Materials Table S2), where the most prevalent were having suffered from family conflicts throughout life, growing up with an introverted personality, and having suffered from bullying at school.

In the self-evaluation aspects, 38.4% felt comfortable with themselves and 50% did not. Regarding work or school aspects, 38.4% of the patients felt fulfilled and 42.3% felt dissatisfied; regarding their affective partners, 26.9% were satisfied and 19.2% dissatisfied. Empathy levels were as follows: only 1 patient (3.8%) showed an “Excellent” level, 17 (65.3%) patients had a range of “Very good”, and 1 person (3.8%) “Good”. Empathy data could not be retrieved from the psychological records of 7 patients.

#### 3.4.2. Hamilton Scales

**Figure 4:**
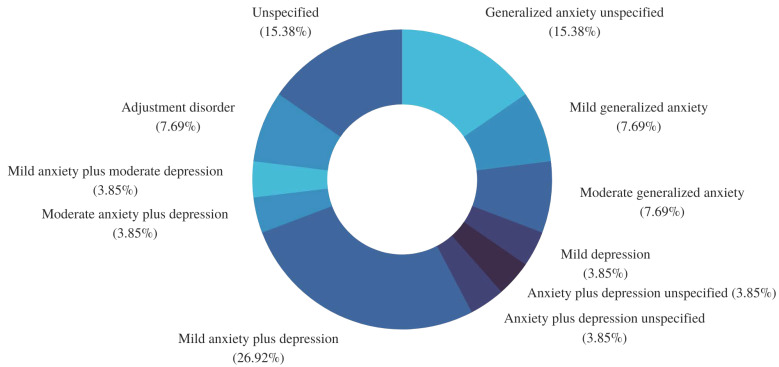
Frequency of psychological diagnoses obtained during the study. Each fraction of the plot represents a percentage calculated from the total number of patients who agreed to and received psychological care (*n* = 26). There were four patients who were categorized as unspecified because they did not complete the intervention, and therefore their diagnostic impression could not be integrated.

During the evaluation of the Hamilton anxiety score, two patients obtained a mild score (7.6%), eight (30.7%) moderate, six (23%) severe, and six (23%) very severe, whereas four (15.3%) did not continue psychological therapy and therefore did not complete this scale. In the evaluation of the scores of the Hamilton depression scale, 1 patient (3.8%) obtained a mild score, 5 (19.2%) moderate, 2 (7.6%) severe, and 13 (50%) very severe; 5 patients did not complete this scale (19.2%). Once the psychological history and the interview evaluation were integrated, the psychologist issued the following diagnostic impressions (Figure 4): the most frequent diagnosis was anxiety plus mild depression, present in 7 patients, followed by unspecified generalized anxiety (*n* = 4). Of the 26 patients who attended psychological treatment, 6 were referred to psychiatry and only 5 accepted it.

### 3.5. Psychiatric Care

From the data collected from the psychiatric clinical records, there were four patients with a family history of mental disorders: in two of them, their mother had depression, the mother of one patient had anxiety, and the mother and cousin of another patient had a diagnosis of schizophrenia. Referring to their current mental health condition, 100% had a long evolution, mostly with onset in childhood. Two patients had polycystic ovary syndrome (PCOS) and one patient had a hyperactive bladder. The most prominent signs observed were as follows: four patients presented with anguish, three patients with despair, two presented with anhedonia, two had a history of self-harm, and two patients had insomnia. Among the symptoms observed in these participants were tachycardia, diaphoresis, tremors, chest tightness, tension headaches, and paresthesia.

Two patients were diagnosed with mixed anxiety–depressive disorder, two with generalized anxiety disorder, and one patient with depressive episodes (Table 2). The treatment used was based on selective serotonin reuptake inhibitors, mainly using fluoxetine, in some cases combined with benzodiazepines and muscle relaxants for accompanying symptoms such as insomnia and bruxism. They are currently in follow-up in conjunction with psychological therapy. Table 2 shows, in a comparative manner, the diagnoses obtained via the three attention levels.

### 3.6. Evaluation of Mentali as a Tool for Primary Screening of Anxiety and Depression

Once the data analysis was completed, a total of 24 patients had completed all items, which allowed us to assess Mentali’s usefulness. For this analysis, we considered only the diagnostic impression of psychology as the gold standard. Two patients were omitted from this analysis because they did not complete the psychological intervention. According to the data described previously, for anxiety, Mentali has a sensitivity of 95% and a specificity of 25%. Regarding depression, the sensitivity and specificity of the app were calculated as 43% and 80%, respectively. The PPV and NPV were 86.4% and 50% in classifying anxiety, and 75% and 50% in classifying depression. For the detection of a mental health disorder, the sensitivity of Mentali was calculated to be 100%.

### 3.7. Mentali as a Tool for Monitoring Mental Health Conditions and Mood during School Periods

**Figure 5:**
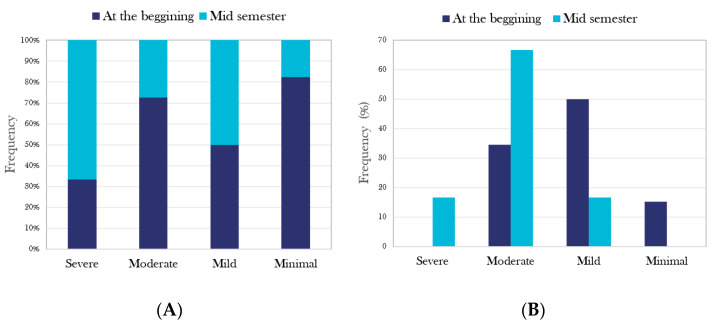
Evolution of the frequencies of anxiety and depression in medical students at two times during the school period. Considering the number of Beck inventories completed by the Mentali users at the beginning and at the middle of the semester, the proportions of positive screenings were considered to construct plots both for depression (**A**) and anxiety (**B**), stratified according to their severity.

In order to evaluate whether the academic periods influenced the mood of the students, three times during the school period were selected considering the academic demands to which students are regularly exposed. The times selected were at the beginning of the academic course, during the first midterm, and during the period of the final academic tests (Table 3). At the beginning of the semester, the users of the Mentali mood module created a total of 310 records; 78.7% of the users claimed to feel excellent/good and 7.4% stated that their mood was bad/very bad. The proportions of users with excellent/good mood decreased from 78.7% to 34.4% at the end of the semester (*p* ≤ 0.001; OR = 0.1, 95% IC: 0.06–0.32). Conversely, the proportion of users who claimed to have bad/very bad mood at the beginning of the semester increased from 7.4% to 34.4% at the end of the semester (*p* ≤ 0.001; OR = 6.5, 95% IC: 2.73–15.76). For anxiety and depression analysis and its evolution during the academic period using the Mentali app, we only selected two times during the semester, because the Beck inventories required at least two positive screenings; at the end of the study, there were few inventories completed by the users. The two times selected were at the beginning of the academic period and at the middle of the semester. Figure 5 shows the results of the comparison of the proportion of positive screenings obtained at the beginning and at the middle of the semester for both depression (Figure 5A) and anxiety (Figure 5B) using Mentali. The proportion of positive screenings for severe depression was 10.3% at the beginning of the semester, and it increased to 37.5% at mid-semester (*p* = 0.05; OR = 5.2, 95% IC: 1.08–24.89). Similarly, there was an increment in severe/moderate anxiety-positive screenings from 34.6% at the beginning to 83.3% at mid-semester (*p* = 0.06; OR = 9.4, 95% IC: 0.95–93.63).

## 4. Discussion

Although it has been reported that one in three medical students suffer from anxiety and approximately 41% experience depression, there are no clear common risk factors associated with these mental conditions in this population [12,13,14]. Additionally, the demands brought about by the COVID-19 health crisis necessitate the implementation of new strategies that allow healthcare professionals to intervene in a timelier manner by leveraging technology. Accordingly, the aim of this study was to evaluate the usefulness of a mobile app (Mentali) as a primary screening tool for anxiety and depressive disorders in Mexican medical students and to evaluate the main factors associated with the development of mental health problems in the studied population.

According to the Mentali primary screening, during the period evaluated, symptoms of moderate/severe anxiety and depression were present in 62.6% and 54.6% of the studied population, respectively. These results are much higher than those reported in the Mexican ENBIARE survey, where it was found that 31.3% and 15.4% of the adult population had symptoms of anxiety and depression, respectively [4]. These findings corroborate the notion that medical students have additional risk factors that predispose them to developing these mental conditions. In spite of the high percentage of Mentali users with symptoms of anxiety and/or depression during the general primary screening, considering the criteria of the Beck inventories for anxiety and depression, only 20% and 8.3% of the users generated alerts that indicated that they should be referred to psychological care. These frequencies are even higher than those reported previously by Ana M. Bassols et al. in a study carried out in Rio Grande do Sul, Brazil [42], and are also similar to those reported by ENBIARE (19.3% for severe anxiety) for the adult population in Mexico [4]. According to the above, the need for the establishment of population-based screenings for the identification and awareness of mental disorders in young adults, including medical students, is highly evident.

At the time of referral to psychology and psychiatry, 65% of the Mentali users accepted the therapy. Previous data reported that among the patients who required psychological care, only 35% visited the mental health specialist to receive the therapy [43]. These results are very important because Mentali allowed us to increase the proportion of patients who needed psychological care and received it by 30%. Of note, among the patients who accepted the therapy, the presence of a mental health disorder was corroborated in 100% of them, which lends credibility to the usefulness of the application for primary screening.

From the data collected, it was shown that 76.9% of the students evaluated in terms of psychology had suffered from family conflicts and feelings of parental disapproval; moreover, 22.9% belonged to a non-traditional family, the main modality being the single-parent family. Additionally, of the patients referred to psychiatry, 80% of them had family antecedents of anxiety and depression. As seen in previous studies, stress, especially that which occurs in the first years of life, is one of the main triggers related to depressive disorders [44]. In addition, emotional, physical, or sexual abuse, family violence, parental delinquency or mental illness, substance abuse [45], and the loss or separation of a parent and lack of attachment are often determinants of children’s mental health [46,47]. The family APGAR is useful for investigating the functioning of a family at a given time [32]. Our study identified 26.8% of patients with some degree of family dysfunction. The family has been considered a strategic nucleus within the psychosocial care model [47], being the main area of implementation of therapeutic interventions [48,49]. On the other hand, maintaining a healthy lifestyle is essential to maintaining mental health [50]. Studies conducted during the COVID-19 pandemic with this approach revealed that of the seven lifestyle domains (diet and nutrition, substance abuse, physical activity, stress management, restful sleep, social support, and environmental exposures), the most affected were exposure to outdoor time (93.6%) and physical activity (70.2%) [51]. Moreover, inappropriate eating habits and body image concerns increased, especially in individuals with pre-existing mental disorders [52]. Regarding the Mentali population who received psychological care, in the long term, 22.9% presented with obesity, 7.6% with bulimia/anorexia, and 15.2% with gastrointestinal problems. In this sense, it is important to note that the role of adipose tissue in controlling emotions has emerged, because it is considered an endocrine organ that secretes peptide hormones that are regulators of metabolism and behavior, including in the brain [53]. Leptin has been related to antidepressant and anxiolytic effects [54], but in the presence of obesity, there is a resistance to it [55], despite being found in the circulation proportionally to the size of the fat mass [56]. Similarly, there is resistance to other adipokines, insulin resistance, and altered plasma cortisol levels, along with hypothalamic–pituitary–adrenal (HPA) dysregulation, also involved in the control of emotions [53]. The accumulation of central fat also stimulates the entry of saturated free fatty acids into the brain, and the release of cytokines and inflammatory signals (C-reactive protein), which may promote neuroinflammatory responses and depressive behaviors. In parallel is the negative affect associated with the self-perception of this condition [57,58]. These pathophysiological changes are also associated with increased suicidal thoughts [58,59,60]; conversely, dietary intervention appears to be a promising therapy to treat these disorders [61,62].

Among other triggering factors, all the patients in psychological therapy stated that they had suffered from bullying at some time in their lives, being more accentuated during infancy, and they presented with problems in socializing. In the school setting, the triggering factor was perceived stress upon entering university, where a lack of concentration had a greater impact [63]. Regarding the DSM-5 criteria, in our study, the most frequent symptoms were low self-esteem, lack of concentration, tiredness, and feelings of guilt. In the self-evaluation, the introversion trait was the most frequent; 50% of the patients did not feel at ease with themselves and 42% were dissatisfied with their school environment, but they did have high levels of empathy. Previous studies have shown that personality traits such as introversion and empathy are risk factors for mental disorder development [64,65].

It is important to highlight that when analyzing the diagnostic capacity [22] of Mentali, a small sample was processed that considered only the patients who fulfilled all the requirements. However, the existence of a mental disorder was evidenced in 100% of the patients referred to psychology and psychiatry, which proves that Mentali is a practical and reliable tool for the screening of mental disorders. The sensitivity and specificity of Mentali to classify anxiety and depression were calculated to be 95% and 43%, and 25% and 80%, respectively. However, it is important to mention that because only the patients undergoing psychological evaluation were considered, the capacity of the mobile application to classify individuals without a condition (specificity) may introduce bias and therefore should be considered cautiously.

The evaluation of the Mentali app as a tool for monitoring the evolution of mood and mental health disorders and their impact during different times of the school period showed differences in the mood of the students at the beginning of the semester and that observed at the final examinations, with increased severity, finding higher bad/very bad responses in 27% of participants. Similarly, the results of the Beck inventories showed that anxiety was associated with an increase of 48.7% in moderate/severe responses at the beginning of exams compared to the beginning of the semester. An increase of 18.3% in moderate/severe responses to the depression inventories was also observed, attributing the increase in severity to the academic demands of the period, as reported previously [66]. These findings are important because they demonstrate that there is a direct influence of academic activities on medical students throughout the semester, which has an important impact on their mental health, and therefore university authorities should implement coping strategies to lessen these effects and create a healthier academic environment.

Finally, our results also showed that 77.1% of the users who installed Mentali interacted with it. However, there was a considerable percentage of the population (89.6%) that interacted with the mood module. Among participants interacting with the mood module, for 40%, this was the only module that they used, leaving the inventories aside. The inventories in general were only used by 60% of the users, which, we assume, may be due to the demand and time involved in answering the questionnaires with numerous items, and it reflects the need to develop new screening tests that are more attractive for young adults at high risk of suffering from anxiety and/or depression.

### Limitations

The main limitation of the study was the number of users included in the evaluation of the classificatory capacity of the app for anxiety and depression. In this study stage, only the patients undergoing psychological evaluation were considered during the calculations, and therefore the capacity of the mobile application to classify individuals without a mental health condition (specificity) may introduce bias and should be considered cautiously. Additional studies will be necessary to reproduce the results and evaluate the diagnostic parameters of Mentali on larger populations. In the same way, in this study only first-semester medical students were considered and therefore the triggering factors of a mental health condition and the associations between mood and academic activities should be considered in that context.

## 5. Conclusions

Depression and anxiety are an important public health problem due to their increased prevalence and strong impact on rates of disability. This study showed that the use of the Mentali mobile application had a good impact on identifying people with a mental health disorder, with a sensitivity of 100%, 95% for detecting anxiety, and 43% for depression. The mobile application showed efficacy as a tool for coordinating the provision of specialized interventions, allowing us to increase the proportion of patients who needed psychological care and received it by 30%. Mentali also demonstrated that there was a direct influence of the academic activities on medical students throughout the semester, which had an important impact on their mood. This information may be used by university authorities to implement coping strategies to lessen the negative effects and to create healthier academic environments. Due to the confinements and limitations of face-to-face activities during the COVID-19 pandemic, the introduction of Mentali for the primary screening of mental health disorders in young adults, including medical students, is useful for diminishing the negative impacts that they bring.

## 6. Patents

The web application was registered with the Mexican Instituto Nacional del Derecho de Autor on 4 January 2022 (Registration number: 03-2021-121414575000-01). Likewise, the registration of the mobile application was made on 10 January 2022 (Registration number: 431 03-2021-090711134000-01).

## Figures and Tables

**Table 1:** Description of triggering factors for anxiety and/or depression by social area.

Variable	Factor	*n* (%)
Family	Conflicts	12 (46.1)
	Disapproval	3 (11.5)
	Absence of relative	3 (11.5)
	Distant relationship	1 (3.8)
	Paternal alcoholism	1 (3.8)
	Unknown	6 (23)
Couples	Conflicts	4 (15.3)
	Distrust	2 (7.6)
	Lack of attention	1 (3.8)
	Lack of communication	1 (3.8)
	Transgender couple	1 (3.8)
	Unknown	17 (65)
Friendship	Couple estrangement	4 (15.3)
	Trouble socializing	2 (7.6)
	Lack of support	1 (3.8)
	Unknown	19 (73)
School	Stress	13 (50)
	Lack of concentration	3 (11.5)
	Frustration	2 (7.6)
	Favoritism	1 (3.8)
	Insecurity about choice career	1 (3.8)
	Discouragement	1 (3.8)
	Unknown	5 (19.2)
Physical health	Weight gain	2 (7.6)
	Polycystic Ovary Syndrome	2 (7.6)
	Anemia	1 (3.8)
	Appendicitis	1 (3.8)
	Hypochondria	1 (3.8)
	Irritable Bowel Syndrome	1 (3.8)
	Unknown	18 (69.2)

**Table 2:** Summary of diagnoses of mental health conditions obtained using Mentali, and psychological and psychiatric evaluations.

User	Diagnosis		
	Mentali	Psychology	Psychiatry
1	Severe anxiety and severe depression	Generalized anxiety and mild depression	Mixed anxious–depressive disorder
2	Severe anxiety and moderate depression	Generalized anxiety	Generalized anxiety disorder
3	Severe anxiety and mild depression	Moderate depression	Moderate depressive episode
4	Severe anxiety and moderate depression	Generalized anxiety	Generalized anxiety disorder
5	Severe anxiety	Generalized anxiety	Generalized anxiety disorder and moderate depressive episode

Five patients who were referred for psychological care were also provided with psychiatric care. This table displays the suspected diagnosis issued by Mentali and the diagnostic corroboration by specialists in psychology and psychiatry.

**Table 3:** Comparisons of mood between different school periods.

Time of Evaluation	Mood Report, n (%)					Total Responses
	Excellent ^a^	Good ^b^	Regular ^c^	Bad ^d^	Very Bad ^e^	
Beginning of the semester	114 (36.7)	130 (41.3)	43 (13.8)	15 (4.8)	8 (2.5)	310
First academic test	12 (4.6)	72 (27.9)	102 (39.5)	44 (17)	28 (10.8)	258
Final academic test	5 (17.2)	5 (17.2)	9 (31.0)	5 (17.2)	5 (17.2)	29

*p*-values < 0.001 for the comparisons between proportions of a vs. e, a vs. d, b vs. e, b vs. d, and a + b vs. d + e. The mood responses provided by Mentali users in three specific periods during the semester with different academic demands (at beginning, during the first academic test, and during the final tests) were quantified and the proportions obtained from the mood results were compared. There was a significant relationship between mood and the school period.

## Data Availability

All data supporting the reported results are included in the manuscript. Additional information regarding data that support the findings of this study will be available from the corresponding author [M.L.M.-F.], upon reasonable request.

## Supplementary Materials

The following supporting information can be downloaded at: https://www.mdpi.com/article/10.3390/brainsci12091223/s1, Table S1: Words of Alarm, Table S2: Description of triggering factors associated with anxiety and/or depression by stage of life.
