# Supplementary material for: Usefulness of a Mobile Application (Mentali) for Anxiety and Depression Screening in Medical Students and Description of the Associated Triggering Factors
Source: Brain Sci. 2022 Sep 10;12(9):1223. doi: 10.3390/brainsci12091223 (PMC9496953; doi:10.3390/brainsci12091223)
Supplement: Supplementary file 1 [file brainsci-12-01223-s001.zip › Table S1.pdf]

Supplementary Table S1. Words of Alarm

Very High		High		Half		Low	
**Word in English**	**Word in Spanish**	**Word in English**	**Word in Spanish**	**Word in English**	**Word in Spanish**	**Word in English**	**Word in Spanish**
Help	Ayuda	Distressed	Angustiado	Exhausted	Agotada	Agitated	Agitado
Crisis	Crisis	Distressed	Angustiada	Exhausted	Agotado	Agitated	Agitada
Blow	Explotar	Anxiety	Ansiedad	Spoiled	Arruinado	Confused	Confundida
Fed up	Harto	Anxious	Ansioso	Spoiled	Arruinada	Confused	Confundido
Fed up	Harta	Anxious	Ansiosa	Spoil	Arruinar	Confused	Confusión
Fed up	Hartada	Attack	Ataque	Tired	Cansado	Too much	Demasiado
Fed up	Hartado	Blame	Culpa	Tired	Cansada	Doctor	Doctor
Awful	Horrible	Guilty	Culpable	Weak	Débil	Doctor	Doctora
Unstable	Inestable	Discouragement	Desaliento	Disappointment	Decepción	Studying	Estudiando
Try	Intento	Tired	Desganado	Upset	Decepcionada	Study	Estudio
Bad	Mal	Pain	Dolor	Devils	Diablos	Homework	Tarea
Terrible	Fatal	Hurts	Duele	Distracted	Distraído	Tense	Tenso
Cry	Llorar	Stress	Estrés	Distracted	Distraída	Terror	Terror
Crying	Llorando	Stressed	Estresado	Sick	Enferma	Out of place	Fuera de lugar
Bad	Malo	Stressed	Estresada	Sick	Enfermo	Until now I had time	Hasta ahorita tuve tiempo
Hate	Odio	Stressful	Estresante	Tired	Fatigado	I will not reach	No alcanzaré
Worse	Peor	I stressed	Estresé	Tired	Fatigada	I can not reach	No alcanzar
Whore	Puta	Encroach	Invadir	Frustration	Frustración	I don't pay attention	No pongo atención
Whore	Puto	Invade me	Invadirme	Impatient	Impaciente	I can't pay attention	No puedo poner atención
Kill myself	Suicidarme	Irritable	Irritable	Incomprehension	Incomprensión	I can´t decide	No puedo decidirme
Suicide	Suicidio	Melancholic	Melancólico	Insecure	Inseguro	I can´t decide	No puedo decidir
Suicide	Suicidar	Melancholic	Melancólica	Insecure	Insegura		
Fuck	Verga	Nerves	Nervios	Unsafety	Inseguridad		
Shit	Mierda	Nervous	Nerviosa	Fight	Pelea		
Defeated	Vencido	Nervous	Nervioso	Lose	Perder		
Alone	Solo	Nostalgia	Nostalgia	I missed	Perdí		
I can´t control myself	No me puedo controlar	Resigned	Resignado	Remorse	Remordimiento		
I can´t control it	No lo puedo controlar	Loneliness	Soledad	I failed a subject	Reprobé		
I am useless	No sirvo para nada	Alone	Solo	Fail	Reprobar		
Kill me	Matarme	Sad	Triste	I will fail	Reprobaré		
Die	Morirme	Sadness	Tristeza	Fear	Temor		
I don´t want to live	No quiero vivir	:(	:(	Fear	Miedo		
I don´t wanna live	Sin ganas de vivir	:c	:c	Mental block	Bloqueo mental		
Abyss	Abismo	Low self-esteem	Baja autoestima	Cost	Cuesta		
I can´t anymore	Ya no puedo más	I don't like my appearance	Mi aspecto no me gusta	I fight	Peleo		
I give up	Me doy por vencido	I am worried	Preocupo	Less friends	Menos amigos		
		Concern	Preocupación	I did not sleep	No dormí		
		I am worried	Preocupan	I'm not doing nothing	No hago nada		
		It consumes	Consume	I haven't been able to sleep	No he podido dormir		
		Worries	Preocupa	I can´t concentrate	No me concentro		
		I still feel the same	Me sigo sintiendo igual	I don't like being with people	No me gusta estar con la gente		
		Nobody loves me	Nadie me quiere	I can't sleep	No puedo dormir		
		I'm not done	No estoy hecho	I don´t want to do	No quiero hacer		
		I'm not done	No estoy hecha	I don´t have money	No tengo dinero		
		Tired	Sin ganas de nada	Lose calm	Perder la calma		
		I don't like how I am	No me gusta como soy	Family problems	Problemas familiares		
		I don't know who to tell	No sé a quién decirle	Low rating	Saldré bajo		
		I do not have friends	No tengo amigos	No strength	Sin fuerza		
		I'm not hungry	No tengo apetito	Criticism	Critica		
		I dont feel like eating	No tengo ganas de comer				
		I'm not hungry	No tengo hambre				
		I never have fun	Nunca me divierto				
		Nothing will ever work out for me	Nunca me saldrá algo bien				
		I lose control	Pierdo el control				
		Without energy	Sin energía				
		Ugly	Feo				
		I do everything wrong	Todo lo hago mal				
		I don't know how to continue	No sé cómo seguir				
		I don't know what to do	No sé qué hacer				

NOTE: Alarm words and digits catalogued by severity
