# Supplementary material for: Usefulness of a Mobile Application (Mentali) for Anxiety and Depression Screening in Medical Students and Description of the Associated Triggering Factors
Source: Brain Sci. 2022 Sep 10;12(9):1223. doi: 10.3390/brainsci12091223 (PMC9496953; doi:10.3390/brainsci12091223)
Supplement: Supplementary file 1 [file brainsci-12-01223-s001.zip › Table S2.pdf]

Supplementary Table S2. Description of triggering factors associated with anxiety and/or depression by stage of life (n = 26).

Aspect	Stage of life, n (%)					
	INFANCY		CHILDHOOD		ADOLESCENCE	
**Family**	Absent father	3 (11.5)	Domestic violence	3 (11.5)	Family distancing	9 (34.6)
	Dysfunctional family	2 (7.6)	Close family death	2 (7.6)	Grandfather's death	3 (11.5)
	They don't remember	2 (7.6)	Sibling separation	2 (7.6)	Homosexual statement	1 (3.8)
	Change of address	2 (7.6)	Sibling care	3 (11.5)	Feelings of guilt	2 (7.6)
	Complicated situations	1 (3.8)	Bullying	1 (3.8)	Rebellion	1 (3.8)
	Unknown	16 (61.5)	Change of house	2 (7.6)	Father's illness	1 (3.8)
			Stress	2 (7.6)	Kidnapping	1 (3.8)
			Unknown	11 (42.3)	Unknown	8 (30.7)
**School**	Bullying	1 (3.8)	Bullying	7 (26.9)	Bullying	4 (15.3)
	They forgot to pick him up from school	1 (3.8)	He did not like it	1 (3.8)	Low grades	4 (15.3)
	Misbehavior	1 (3.8)	Internship	1 (3.8)	Internship	1 (3.8)
	Introvert	1 (3.8)	Unknown	17 (65.3)	Behavior problems	2 (7.6)
	Hard	2 (7.6)			Unknown	15 (57.6)
	Unknown	20 (76.9)				
**Friendship**	Few friends	5 (19.2)	Few friends	10 (38.4)	Few friends	3 (11.5)
	Bullying	1 (3.8)	Unknown	16 (61.5)	Difficulty socializing	2 (7.6)
	Unknown	20 (76.9)			Feelings of loneliness	1 (3.8)
					Unknown	20 (76.9)
**Couples**	*	—	*	—	Bad relationship	2 (7.6)
					Unknown	24 (92.3)
**Work**	*	—	*	—	Work	1 (3.8)
**Physical health**	Over weight	2 (7.6)	Overweight	4 (15.3)	Bulimia and anorexia	2 (7.6)
	Neonatal hypoxia	2 (7.6)	Accidents	3 (11.5)	Gastritis	2 (7.6)
	Run over	1 (3.8)	Frequent hospitalizations	1 (3.8)	Irritable Bowel Syndrome	1 (3.8)
	Malnutrition	1 (3.8)	Appendectomy	1 (3.8)	Migraine	2 (7.6)
	Asthma	1 (3.8)	Anemia	1 (3.8)	Broken arm	1 (3.8)
	Surgery	1 (3.8)	Vertigo	1 (3.8)	Cutting	1 (3.8)
	Allergy	1 (3.8)	Irritable Bowel Syndrome	1 (3.8)	Eating disorders	1 (3.8)
	Neck injury	1 (3.8)	Myopia	1 (3.8)	Unknown	16 (61.5)
	Accident	1 (3.8)	Unknown	13 (50)		
	Unknown	15 (57.6)				
**Personality traits**	Introvert	7 (26.9)	Introvert	9 (34.6)	Introvert	8 (30.7)
	Outgoing	1 (3.8)	Outgoing	2 (7.6)	Glad	2 (7.6)
	Calm	2 (7.6)	Irritable	1 (3.8)	Obsessive	2 (7.6)
	Restless	2 (7.6)	Obsessive	1 (3.8)	Antisocial	1 (3.8)
	Skilled	1 (3.8)	Cheerful	1 (3.8)	Unknown	13 (50)
	Easy crying	1 (3.8)	Unknown	12 (46.1)		
	Unknown	12 (46.1)				

* The features does not apply. Unknown: Participants who did not complete the section and therefore the information was not available.
